# The Role of Feedback Loops in Targeted Therapy for Pancreatic Cancer

**DOI:** 10.3389/fonc.2022.800140

**Published:** 2022-05-16

**Authors:** Weigang Gu, HongZhang Shen, Lu Xie, Xiaofeng Zhang, Jianfeng Yang

**Affiliations:** ^1^Department of Gastroenterology, Affiliated Hangzhou First People’s Hospital, Zhejiang University School of Medicine, Hangzhou, China; ^2^Key Laboratory of Integrated Traditional Chinese and Western Medicine for Biliary and Pancreatic Diseases of Zhejiang Province, Hangzhou, China

**Keywords:** pancreatic cancer, feedback loops, targeted therapy, negative regulation, positive regulation

## Abstract

Pancreatic cancer is the leading cause of cancer-related deaths worldwide, with limited treatment options and low long-term survival rates. The complex and variable signal regulation networks are one of the important reasons why it is difficult for pancreatic cancer to develop precise targeted therapy drugs. Numerous studies have associated feedback loop regulation with the development and therapeutic response of cancers including pancreatic cancer. Therefore, we review researches on the role of feedback loops in the progression of pancreatic cancer, and summarize the connection between feedback loops and several signaling pathways in pancreatic cancer, as well as recent advances in the intervention of feedback loops in pancreatic cancer treatment, highlighting the potential of capitalizing on feedback loops modulation in targeted therapy for pancreatic cancer.

## Introduction

Pancreatic cancer is the most common type malignant tumor with a high degree of malignancy and poor prognosis, which has now become a major medical dilemma and formidable challenge. Pancreatic ductal adenocarcinoma patients account for more than 90% of pancreatic cancer (1). The individual incidence of pancreatic cancer is about 1.562%, the median survival rate is less than 11 months, and the 5-year survival rate is only 5% (2). Due to its extremely poor prognosis, pancreatic cancer is already the fourth leading cause of cancer-related deaths in western developed countries (2), and it may become the second leading cause of cancer-related deaths within the next ten years (3, 4). Most cases of pancreatic cancer are thought to arise from the production of microscopic precursors called pancreatic intraepithelial neoplasia (5). Surgery is still the main treatment for pancreatic cancer, and this method has evolved from a high-risk operation to a relatively safe operation today (6). However, if surgery alone is not supported by other treatments, most patients will relapse and die of cancer. Sadly, most targeted drugs fail to improve the survival rate of patients for evaluations of treatment alone or combined with chemotherapy (7, 8). Recently, emerging evidence has revealed that the failure of targeted therapies to improve the outcome of advanced pancreatic cancer may be due to signal feedback loops, intermolecular crosstalk and induced drug insensitivity in the disease (9, 10). It is suggested that modulation of feedback loops holds promise for enhancing therapeutic benefits of pancreatic cancer treatment.

The concept of feedback loops is the basis for understanding signal transduction and maintaining homeostasis. There are two essences of feedback regulation, namely negative feedback loop and positive feedback loop, which give different attributes and dynamic characteristics of network dynamics. Inhibiting or activating upstream and downstream signaling proteins will produce clear positive or negative feedback, and sometimes also form feedback loops that act crucial roles in cancer progression (11). Feedback loops have been considered to be involved in important cellular modulated methods that cause autophagy, epithelial-mesenchymal transition, intracellular signaling and hypoxia (12–16). Accumulating evidences show that feedback loop regulation must be directly associated with cancer development, progression and metastasis (17). As one of the solid tumors with high incidence, the signal regulation of pancreatic cancer has been extensively studied and flourishing in recent years. Researchers are interested in the role of feedback loops in the development of pancreatic cancer, but specific roles of feedback crosstalk remain elusive. Here we summarize the feedback loops that exist in related fields, which should play critical roles in the progression of pancreatic cancer and treatment resistance. And it is explained the latest research in the intervention of feedback loops to enhance the therapeutic effect of drugs in pancreatic cancer.

## Positive Feedback Loop

Positive feedback is the basis of powerful dynamic and phenotypic switching in noisy environments, causing conversion from analog input signals to digital outputs and triggering cell fate decisions and phenotypic changes (18). Therefore, we will discuss how multiple protein-protein interactions generate positive feedback loops in the signal transduction cascade and participate in the progression of pancreatic cancer, which will provide enlightenments for the exploitation of drug intervention strategies in pancreatic cancer (**Table 1**).

**Table 1:** The role of different forms of feedback loop in pancreatic cancer.

Feedback loop modulation	Key molecules	Modulation means	Key finding	References
Positive	KRAS/Lin28B/let-7i/TET3	shRNA	Promotes the stemness of pancreatic cancer cells	(19, 20)
Positive	KRAS/E2F1/ILK/hnRNPA1	shRNA, pharmacological inhibition	Promotes oncogenic KRAS signaling to promote aggressive phenotypes	(21, 22)
Positive	KRAS/eIF5A/PEAK1	shRNA, pharmacological inhibition	Attenuates cancer cell growth and migration and block tumor formation in PDAC	(23)
Positive	FAM83/MEK/ERK/JUNB/FOSB	siRNA, shRNA, pharmacological inhibition	Overcomes the obstacles that KRAS is difficult to target and choose other tumor-supressive proteins that are important in RAS signal	(24)
Positive	mutant-P53/STAT3/HSP90	shRNA, pharmacological inhibition	HSP90 and mevalonate pathway sustain the criminal alliance of STAT3 and mutant-P53	(25, 26)
Positive	P53/ALKBH5/PER1	shRNA	ALKBH5 suppresses PC by regulating the posttranscriptional activation of PER1 through m6A abolishment	(27)
Positive	GPR87/JAK2/STAT3	shRNA	Promotes the stemness of pancreatic cancer cells	(28)
Positive	REG3A/JAK2/STAT3	shRNA	Promotes develop,emt of pancreatic cancer cells	(29)
Positive	AGER/IL6/STAT3	Knockout mice	Finds the crosstalk between STAT3 and autophagy	(30)
Positive	PLACT1/hnRNPA1/IκBα/E2F1	siRNA, shRNA, pharmacological inhibition	Facilitates PDAC cells proliferation and invasion	(31)
Positive	MTSS1-AS/MZF1/Myc	siRNA, mimic	Promotes the acidity-metastasis of pancreatic cancer	(32)
Positive	GLS-AS/GLS/Myc	siRNA, mimic	Demostrates the coupling role of lncRNA in nutrient stress and tumorigenesis	(33)
Positive	PTTG3P/miR-132/212-3p/FOXM1	shRNA	Serves as a potential diagnostic marker for clinical treatment	(34)
Positive	HGF/Met/FOXM1	siRNA, pharmacological inhibition	Promotes PDAC development and resistance to Met inhibition.	(9)
Positive	FOXM1/ATX/Hippo	siRNA, lentivirus	Promotes the progression of pancreatic cancer	(35)
Positive	miR-371-5p/ING1	siRNA, mimic	Serves as a potential diagnostic marker for clinical treatment	(36)
Positive	miR-301a/NKRF/NF-κB	siRNA, mimic	Increases NF-κB gene expression and impedes xenograft tumour growth	(37)
Positive	NEAT1/miR-302a-3p/RELA	siRNA, mimic	Facilitates PDAC cells proliferation and invasion	(38)
Positive	Pin1/P65/IL-18	siRNA, lentivirus	Serves as a potential diagnostic marker for clinical treatment	(39)
Positive	miR-135b/BMAL1/YY1	siRNA, mimic	Mediates pancreatic circadian clock disruption	(40, 41)
Positive	miR-30a/SNAI1/IRS1/AKT/FOXO3a	siRNA, mimic	Promotes PDAC development and chemoresistance	(42)
Positive	AKT/BRG1	shRNA, pharmacological inhibition	Promotes PDAC development and chemoresistance	(43)
Positive	HIF1α/P4HA1	siRNA, vector	Demostrates a critical regulator in glycolysis and oncogenic activities of pancreatic cancer	(44)
Positive	LIMS1/AKT-mTOR/HIF1α	shRNA, lentivirus	Facilitates tumor cell adaptation to the glucose deprivation stress	(45)
Positive	ROS/EGFR/MEK/ERK/HIF-1α	siRNA, pharmacological inhibition	Facilitates PDAC cells metastasis and invasion	(46)
Positive	CASC9/AKT/HIF-1α	siRNA, vector	Facilitates glycolytic metabolism and EMT of pancreatic cancer	(47)
Positive	OGT/YAP	siRNA, pharmacological inhibition	Promotes the development of pancreatic cancer cells	(48)
Positive	HIF1α/YAP	siRNA, shRNA	Finds the treatment of YAP inhibitors to improve PC patients with smoking	(49)
Positive	ZEB1/ESRP1/CD44	siRNA, shRNA	Facilitates PDAC cells metastasis and invasion	(50)
Positive	AURKA/Twist1	shRNA, pharmacological inhibition	Promotes EMT and chemoresistance of pancreatic cancer	(51)
Positive	AURKA/ALDH1A1	shRNA, pharmacological inhibition	Facilitates PDAC cells metastasis and invasion	(52)
Positive	TGF-β1/Kindlin-2/TβRI	siRNA, vector	Facilitates PDAC cells metastasis and invasion	(53)
Positive	SDF-1/SATB-1	siRNA, shRNA	Maintains the characteristics of CAF and malignant progression of pancreatic cancer and gemcitabine-resistance	(54)
Positive	CCL18/VCAM-1	siRNA, shRNA	Promotes the development of pancreatic cancer cells	(55)
Positive	DcR3/STAT1/IRF1	siRNA, vector	Promotes the development of pancreatic cancer cells	(56)
Positive	Cav-1/ROS	–	Promotes the growth of pancreatic cance and induced matrix-tumor metabolic coupling	(57)
Negative	HIF1α/miR-646/MIIP/HDAC6	siRNA, vector	Impedes the development of pancreatic cancer cells	(58)
Negative	TWIST1/miR-214/OGDHL/HIF1α/AKT	shRNA, pharmacological inhibition	Impedes PDAC development and metastasis	(59)
Negative	YAP/GLI1/Hedgehog	siRNA, vector	Promotes the development of pancreatic cancer cells	(60)
Negative	DUSP1/MAPK	shRNA, pharmacological inhibition	Promotes the development of pancreatic cancer cells	(61)

### KRAS Signaling

**Figure 1:**
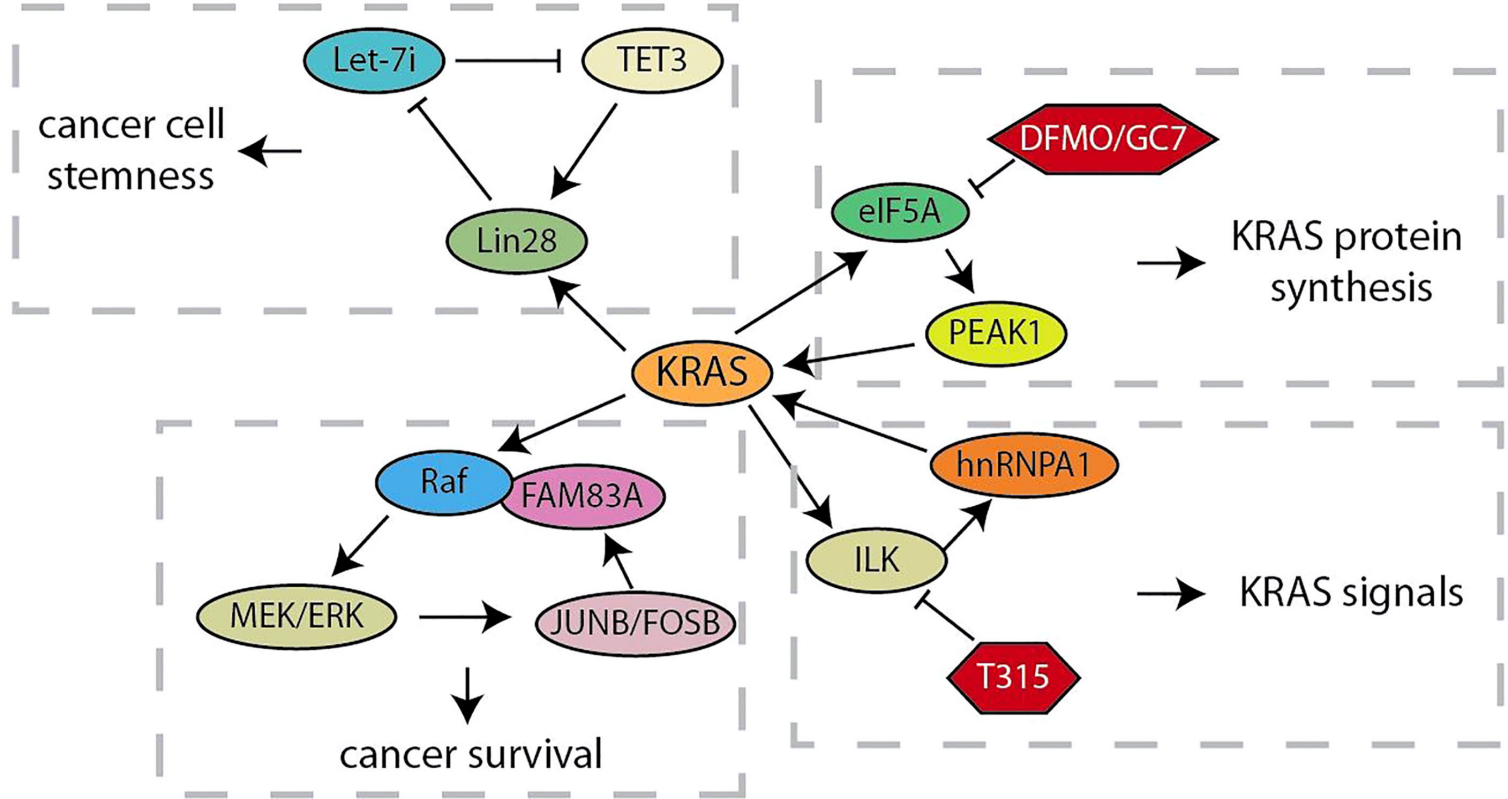
Summary of KRAS related feedback loops in pancreatic cancer.

As an oncogene that can rapidly activate RAF/MEK/ERK and PI3K/AKT/mTOR signals, KRAS continuously activation caused by several mutations drives the development of more than 95% of pancreatic ductal adenocarcinoma (62). Considering that the positive feedback loop is characterized by continuous activation, the research on feedback loops related to KRAS will help us better understand and explore its role in driving pancreatic cancer tumorigenesis (**Figure 1**). Let-7 microRNA expression is selectively blocked by the RNA-binding protein Lin28 to activate downstream targets (such as KRAS, c-Myc, BLIMP1 and HMGA) for maintaining CSC (19). Xu et al. have demonstrated that, the nuclear translocation of Lin28B, highly expressed in pancreatic cancer tissues, is promoted by KRAS through protein kinase C (PKCβ). Subsequently, nuclear Lin28B increases the expression of TET3 by inhibiting mature let-7i. The increased TET3 will activate Lin28B for maintaining the stemness of pancreatic cancer (20). Thus, KRAS maintains the stemness of pancreatic cancer cells through a unique Lin28B/let-7i/TET3 feedback loop. Besides, KRAS signal was also regulated by the KRAS-E2F1-ILK-hnRNPA1 regulatory loop (21, 22). These studies have showed that KRAS regulates the integrin-linked kinase (ILK) through E2F1-mediated transcriptional activation, which regulates the KRAS expression through hnRNPA1-mediated G-quadruplex destabilization on the KRAS promoter. This regulatory loop provides a mechanistic basis for targeting ILK to inhibit oncogenic KRAS signaling. In addition, the oncogenic activation of KRAS hijacks the eIF5A-PEAK1 translation signal transduction, which in turn promotes the accumulation of KRAS (23). This regulatory loop utilizes the unique sustain subtype of the translation elongation factor eIF5A and the tyrosine kinase PEAK1 to increase KRAS protein synthesis in metabolic and energy demands of pancreatic cancer. When some researchers chose to target other tumor effectors that are important in RAS signal transduction in order to overcome the obstacle that KRAS is difficult to be targeted, they found Family with Sequence Similarity 83A (FAM83A) is carcinogenic, exclusive, and similar functions to RAS by driving the activation of PI3K and MAPK signaling (24). The up-regulation of FAM83A maintains the necessary MEK/ERK signals, and JUNB and FOSB activated by MEK/ERK are responsible for the increased expression of oncogenic FAM83A. These studies provide new insights into the progression of RAS-driven pancreatic cancer and lay the foundation for the development of new pancreatic cancer therapies bypassing targeting KRAS.

### STAT3 Signaling

**Figure 2:**
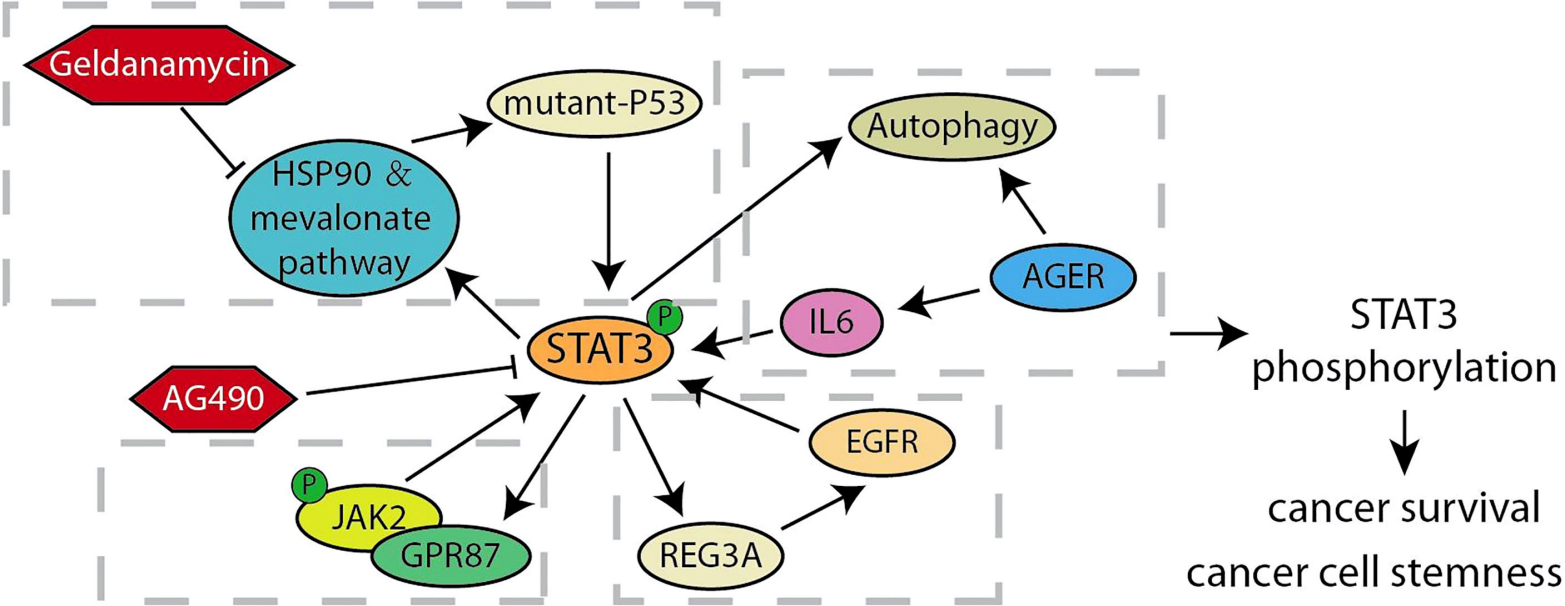
Summary of STAT3 related feedback loops in pancreatic cancer.

Signal transducer and activator of transcription 3 (STAT3) is becoming part of the most prevalent studies in anti-cancer therapy, owing to its inhibition reported to trigger a variety of cell apoptosis in hematologica and solid cancers that show constitutive STAT3 activation (63). As the most well-known tumor suppressor gene, TP53 is suppressed by STAT3 at the transcription level, but the mutation of TP53 in cancer partly eliminates this limitation (64). Recent studies elucidated the interaction between STAT3 and mutp53. The study showed that mutant-P53, unlike wildtype-P53, maintains STAT3 phosphorylation by replacing the phosphatase SHP2 (25). In another study, scholars found that inhibiting STAT3 in pancreatic cancer cells reduces the expression level of mutant-P53 through embaring HSP90 and molecules related to the mevalonate pathway, and the expression of mutant-P53 mediates the phosphorylation of STAT3 in turn (26). These studies revealed that the feedback loop established by mutp53 and STAT3 is a criminal alliance that plays crucial roles in promoting cancer. In particular, TP53 plays a regulatory role in another crosstalk loop (27). ALKBH5 m6A demethylation induced by TP53 activates PER1, and the up-regulation of PER1 leads to reactivation of ATM-CHK2-P53 signaling, thereby inhibiting the growth of pancreatic cancer. During the expansion of pancreatic ductal adenocarcinoma stem cells, the promoter of G protein-coupled receptor 87 (GPR87) is directly bounded by STAT3 to increase its expression. On the contrary, GPR87 activates JAK2/STAT3 to form a positive feedback loop to promote the stemness of pancreatic ductal adenocarcinoma cells (28). Xiang et al. found another similar feedback mechanism. Oncogenic JAK2/STAT3 is activated by exposure to IL-6 in pancreatic cancer cells to directly up-regulates the expression of regenerative gene protein 3A (REG3A), activation of REG3A will instead enhance the JAK2/STAT3 pathway, leading to the amplification of carcinogenic effects of IL-6/JAK2/STAT3 (29). Interestingly, there is also a feedback crosstalk between STAT3 and autophagy. Advanced glycation end product-specific receptor (AGER)-mediated autophagy promotes IL6-induced STAT3 phosphorylation and mitochondrial localization. Moreover, the researchers have observed a positive feedback loop between autophagy activation and IL6-STAT3 pathway, which contributes to our understanding for early precursor lesion development in pancreatic cancer (**Figure 2**) (30).

### lncRNAs

**Figure 3:**
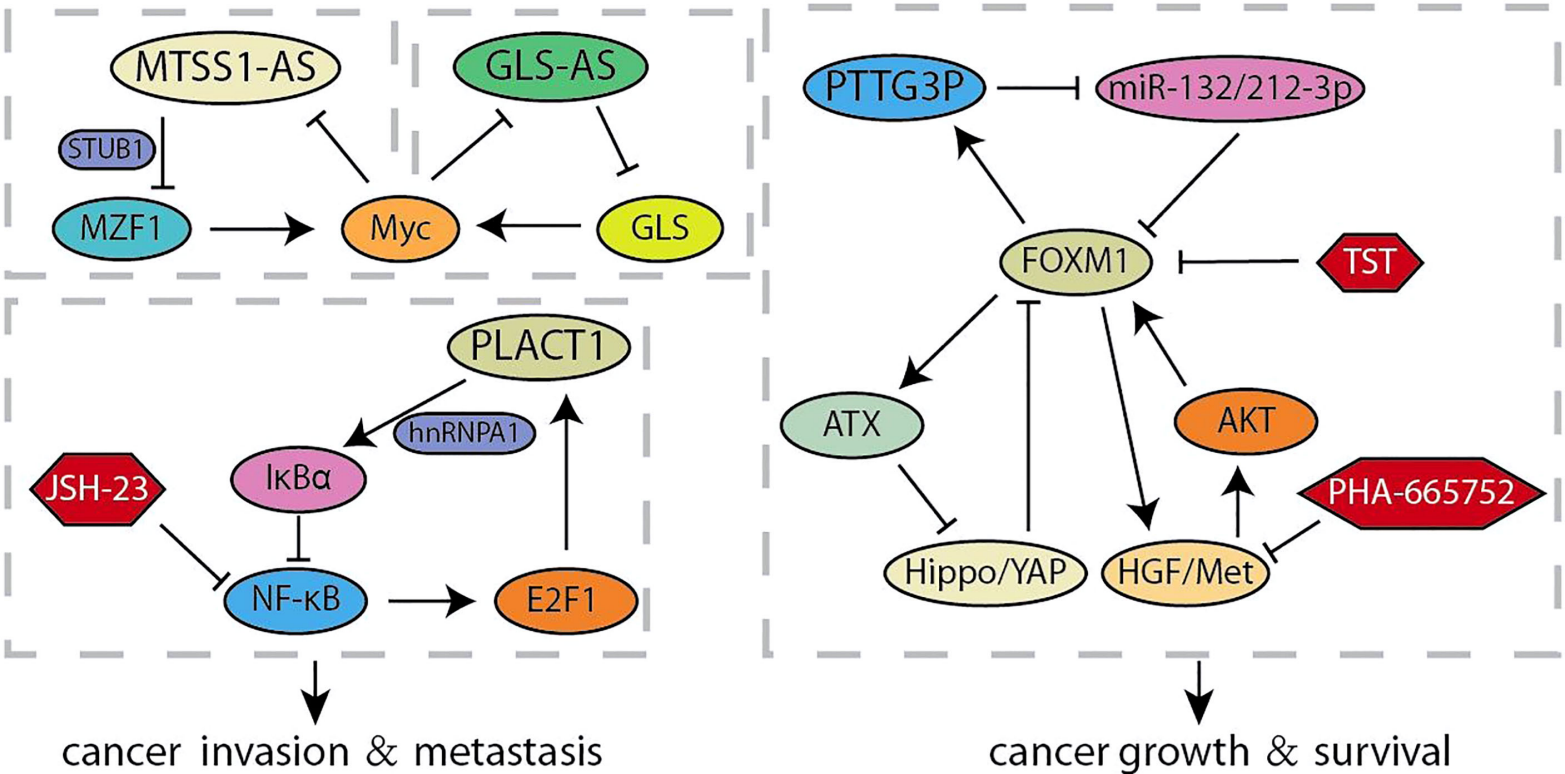
Summary of LncRNA related feedback loops in pancreatic cancer.

Long non-coding RNAs (lncRNA) is a type of RNA that is longer than 200-nt and lacks the potential to encode proteins, which acts multiple roles in the progression of human cancer (65). Accumulating evidence confirmed that lncRNAs are characterized by participating in invasion and metastasis (66, 67). It was reported that a novel lncRNA RP11-1149O23.3, termed as pancreatic cancer associated transcript 1 (PLACT1), inhibited IκBα-E2F1 signaling by recruiting heterogeneous ribonucleoprotein A1 (hnRNPA1) to the promoter region of IκBα. The transcriptional upregulation of PLAT1 mediated by E2F1 continues to activate the NF-κB signaling pathway through forming a positive feedback loop with IκBα, thereby facilitating PDAC cells proliferation and invasion (**Figure 3**) (31). Zhao et al. compared the RNA sequencings of PC cells cultured with different acidity and found that lncRNA (RP11-532M24.1), an antisense RNA of metastasis suppressor 1 termed as lncRNA-MTSS1-AS, was significantly decreased (32). MTSS1-AS supported the interaction between the E3 ubiquitin protein ligase STUB1 and the transcription regulator myeloid zinc finger 1 (MZF1), which leads to its degradation. The impaired MZF1 reduced the transcriptional activation of Myc. Meanwhile, MTSS1-AS was transcriptionally inhibited by the binding of Myc and MTSS1-AS promoter elements, suggesting a reciprocal feedback loop between MTSS1-AS and Myc promoting the acidity-metastasis of pancreatic cancer. Recent results identified an antisense lncRNA of glutaminase (GLS-AS) and demonstrated its link with altered metabolism in pancreatic cancer (33). The expression of GLS was impeded post-transcriptionally by GLS-AS by forming double-stranded RNA with GLS pre-mRNA. Myc activated by nutrient stress decreased the expression of GLS-AS, and GLS-AS reduced conversely the expression of Myc by impeding the GLS-mediated stability of Myc. These results implied a reciprocal feedback loop to allow us to further understand the coupling role of lncRNA in nutrient stress and tumorigenesis. The functional study of Wang et al. revealed that lncRNA Pseudogene pituitary transforming tumor 3 (PTTG3P) prevents its association with FOXM1-mRNA by acting as the ceRNA of miR-132/212-3p. Meanwhile, elevated FOXM1 transcriptionally activated PTTG3P, thereby forming a feedback circuit to enhance the tumor-promoting effect induced by PTTG3P (34). Intriguingly, other articles show other feedback loops in the FOXM1 transcription center. Xie et al. found that activation of HGF/Met signal increases the expression and transcriptional activity of FOXM1, so that more FOXM1 bind to the promoter region of Met gene to enhanced the activation of HGF/Met signal and its downstream pathways. The feedback loop formed by HGF/Met- FOXM1 promotes the growth of PDA and resistance to Met inhibition (9). Other articles demonstrated that ATX is the downstream transcription target gene of FOXM1, and ATX can promote FOXM1 expression by inhibiting the Hippo signaling pathway, thereby promoting the progression of pancreatic cancer (35).

### MicroRNAs

**Figure 4:**
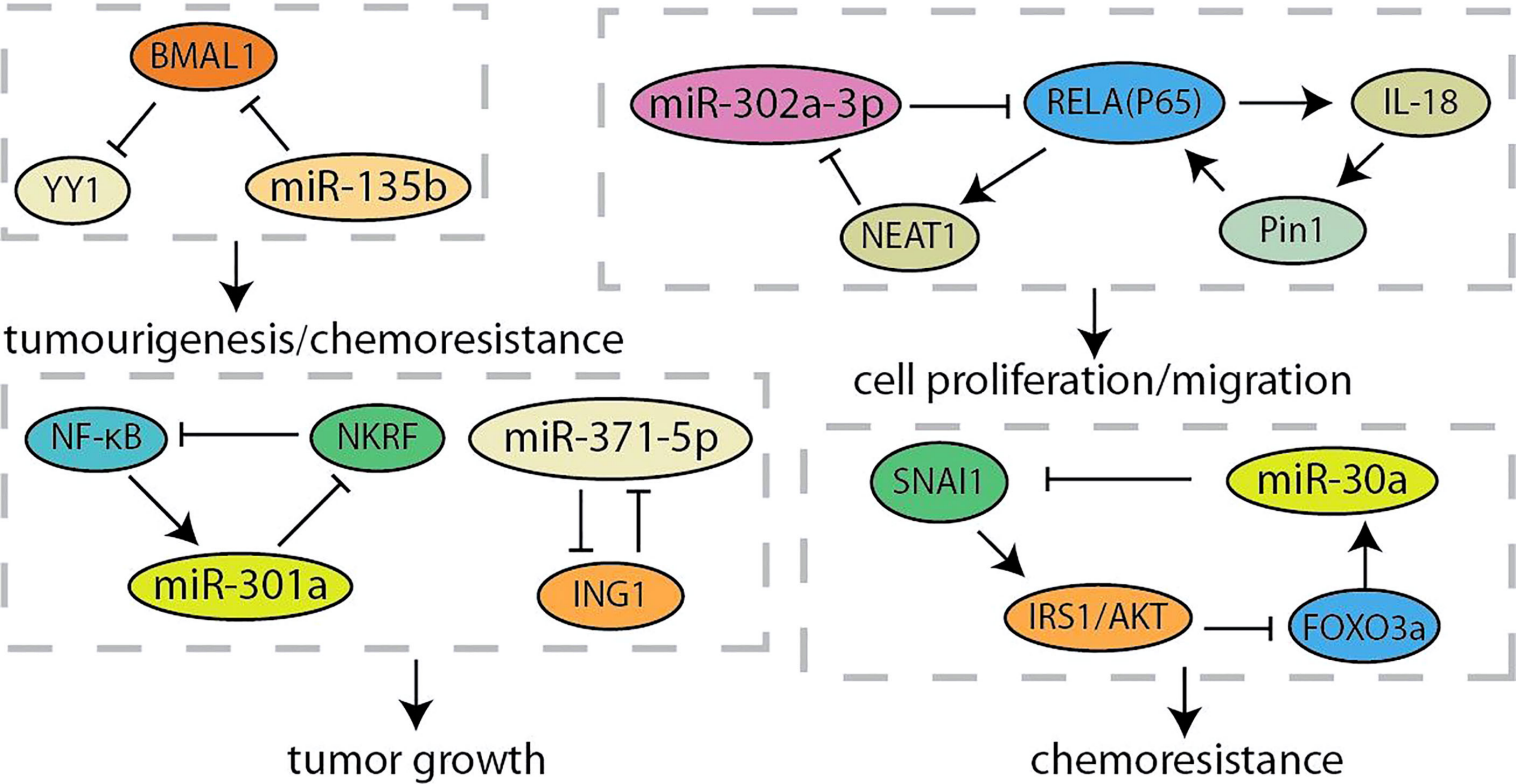
Summary of microRNA related feedback loops in pancreatic cancer.

MicroRNA (miRNA) is a type of RNAs that is a small 21~24-nt non-coding RNA and regulates gene expression by the binding the 3’-UTR of target mRNA to reduce translation or induce mRNA cleavage (68). Thus, the discovery of unknown miRNAs presents a new insight into the cancer research (69). Researchers have found that overexpression of miR-371-5p downregulates growth inhibitor 1 (ING1) and leads to increased pancreatic cancer cells proliferation and tumor growth (**Figure 4**). In turn, ING1 inhibited the expression of miR-371-5p in the promoter region (36). NF-κB has been found to be continuously activated in pancreatic cancer, and regulated by several miRNAs (70, 71). MiR-301a activated NF-κB by negatively regulating the NF-κB inhibitory factor (NKRF). The expression of miR-301a was upregulated when NF-κB is activated, indicating the existence of a continuous positive feedback loop during NF-κB activation (37). According to the recent report, lncRNA-NEAT1/miR-302a regulated the expression of RELA (p65), a component of NF-κB family, in pancreatic cancer. And NEAT1 expression was activated by RELA to modulating cancer metastasis and proliferation (38). Otherwise, the interaction between p65 and Pin1 enhanced NF-κB signaling and activated IL-18, while the increased IL-18 continues to amplify NF-κB signaling, improving the understanding of regulation for NF-κB in pancreatic cancer (39). Yin-Yang 1 (YY1) is a highly conserved zinc finger transcription factor, which has two opposite functions as a transcription repressor or transcription activator during tumorigenesis (40). Accumulating evidences indicated that YY1 regulates tumorigenesis and development by inhibiting the expression of miRNAs (72, 73). YY1 could make more miR-135b targeting at BMAL1 3’-UTR to suppress its expression by directly activating the miR-135b promoter, which formed a “miR-135b–BMAL1–YY1” ring. This disrupted the entire molecular clockwork inside the exocrine pancreas, resulting in weakened circadian control of pancreatic cancer (40, 41). It is reported that YY1 directly regulates the expression of autophagy-related miR-30a. On the contrary, the overexpression of miR-30a attenuates the autophagy-promoting effect of YY1, which means that autophagy in pancreatic cancer may be partly regulated by the coordinated YY1/miR-30a regulatory circuit (74). In particularly, miR-30a was identified as one node of this network that modulated cellular response to gemcitabine through SNAI1-IRS1-AKT pathway. And miR-30a was regulated by AKT-FOXO3a to form a feedback loop (42). Furthermore, AKT was another component of the crosstalk circuit that inhibition of Akt phosphorylation reduces BRG1 expression in pancreatic cancer. The knockdown of BRG1 inhibited the phosphorylation of Akt and decreased acquired chemoresistance to prevent pancreatic cancer development (43).

### Hypoxia and Glucose Metabolism

**Figure 5:**
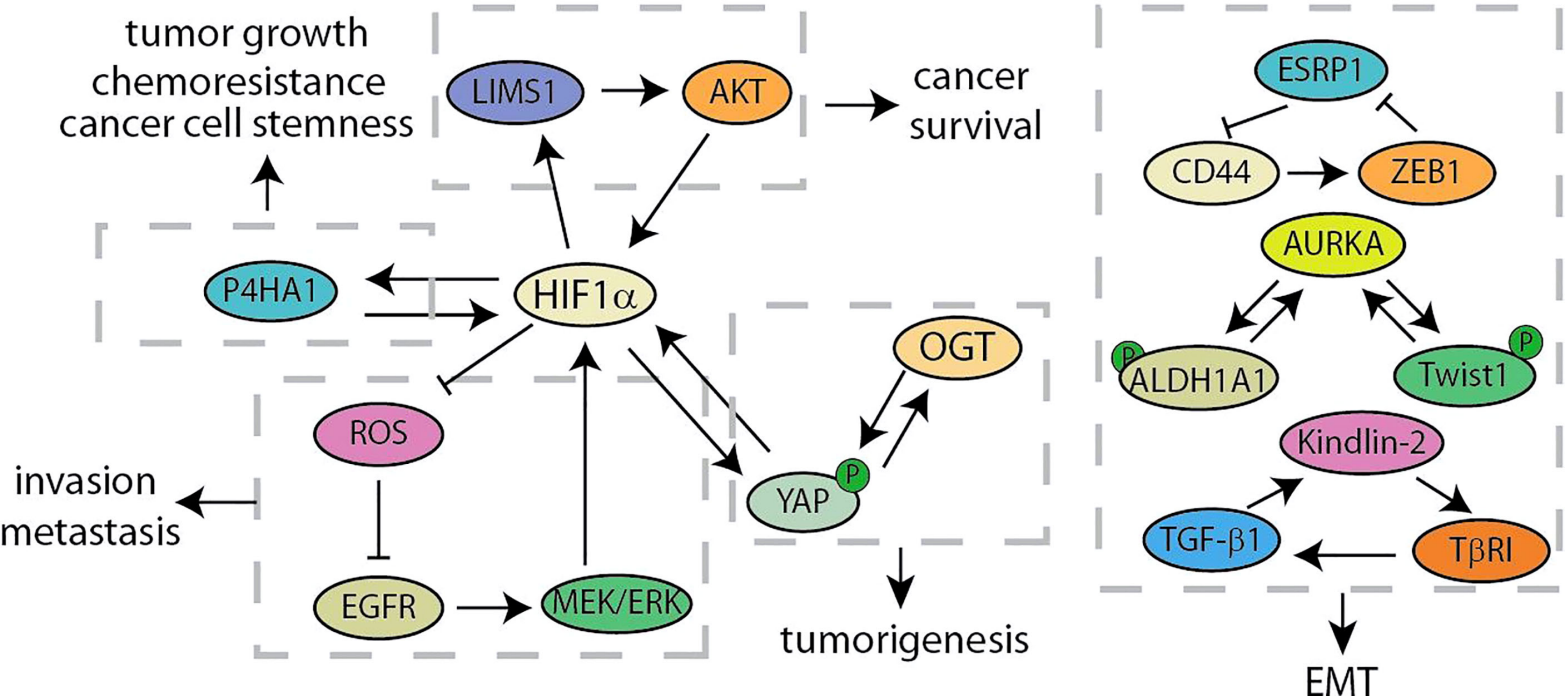
Summary of hypoxia and glucose metabolism and EMT related feedback loops in pancreatic cancer.

Poor tumor vasculature results in the pathophysiological hypoxia in solid tumors, which cause major energy-metabolic differences in the microenvironment between solid tumor tissues and normal tissues. As a result of the Warburg effect, the concentration of glucose in the tumor microenvironment is usually 3 to 10 times lower than that in normal tissues (75, 76). In this section, we have summarized these signal feedback loops related to hypoxia and glucose metabolism to find new clues of developing therapy for pancreatic cancer (**Figure 5**). Glycolysis- and hypoxia-related genes are closely related by using analysis of the dataset from TCGA. According to the above analysis results, Prolyl 4-hydroxylase subunit alpha 1 (P4HA1), elevating in pancreatic cancer cells, is hypoxia-inducible factor 1α (HIF1α)-dependent. Further experimental results showed that P4HA1 can increase the stability of HIF1α to form a positive feedback loop, which is a critical regulator in glycolysis and oncogenic activities of pancreatic cancer (44). LIM and senescent cell antigen-like-containing domain protein 1 (LIMS1) is activated during the oxygen-glucose deprivation stress in PDAC cells. LIMS1 promotes the translation of HIF1α by activating the AKT/mTOR signaling, while HIF1α activates the transcription of LIMS1, thus forming a positive feedback loop for facilitating the adaptation of PDAC cells to the glucose deprivation stress (45). In order to explore the potential molecular mechanism of glucose metabolism related to the invasion and metastasis of pancreatic cancer, Tang et al. screened these genes that related to anaerobic glycolysis regulated by HIF1α (46). Under hypoxic conditions, the EGFR/MEK/ERK pathway activates HIF1α that inhibits the excessive release of ROS, hereafter the reduction of ROS further activates EGFR to form a positive feedback loop. This crosstalk is closely related to the invasion and metastasis ability of pancreatic cancer. Notably, lncRNA CASC9 promotes glycolytic metabolism and EMT in pancreatic cancer through a positive feedback loop with AKT/HIF1α, and hypoxia conditions will synergistically amplify this biological effect (47). In addition, O-GlcNAc transferase (OGT) occupies an important position in the regulation of glycolytic metabolism. OGT modifies YAP at serine 109 to inhibit the interaction between YAP and its upstream kinase, LATS1, thereby activating its transcriptional activity. Besides, OGT has been determined as a gene transcriptionally regulated by YAP, forming a feedback loop with YAP to promote tumorigenesis (48). Recent studies have revealed that the nuclear localization and transactivation of YAP are promoted by HIF1α when treated with nicotine (49). In addition, YAP increased and sustained the protein stability of HIF1α. The development of inhibitors specifically targeting YAP/HIF1α may provide a new strategy for treating PC patients with a history of smoking.

### Epithelial Cell-Mesenchymal Transition

Cancer recurrence and metastasis are the main factors for the failure of clinical treatment programs, which are partly due to epithelial-mesenchymal transition (77, 78). The key signalings of EMT mainly include the ZFH family (ZEB1 and ZEB2), the bHLH family (E12/47 and Twist), the snail family (Snail and Slug) and so on (79). Recent studies have identified a positive feedback loop between CD44 and ZEB1 for supporting the aggressive metastatic phenotype (**Figure 5**) (50). Stemness and mesenchymal characteristics are maintained by the expression of ZEB1 activated by CD44 in pancreatic cancer. ZEB1 in turn inhibits the transcription of the splicing factor ESRP1, leading to enhanced shift of CD44 splicing to favorably express CD44. Shah et al. have discovered the crucial role of Aurora Kinase A (AURKA)-Twist1 axis in promoting EMT and chemoresistance of pancreatic cancer (51). As a AURKA’s EMT specific target, Twist1 can be phosphorylated by AURKA at serine 123, threonine 148 and serine 184 to inhibit its ubiquitination, increase its transcriptional activity and promote its homodimerization. At the same time, Twist1 reciprocates and prevents AURKA from degrading, thereby triggering the positive feedback loop. Besides, another study has shown that AURKA and ALDH1A1 form a similar mechanism of feedback loop (52). AURKA-mediated phosphorylation rapidly dissociates the ALDH1A1 tetramer into highly active monomer. And monomeric ALDH1A1 reciprocates and prevents the degradation of AURKA, further driving a robust expression of the activity of EMT-related factors Snail, Slug and CD44. In addition, it is reported that TGF-β1 decreases HOXB9 and E-cadherin to promote PDAC progression by activating Kindlin-2, while Kindlin-2 upregulates transforming growth factor receptor I (TβRI), a key component of TGF-β signaling, for forming a positive feedback loop (53).

### The Crosstalk of Cells and Tumor Microenvironment

**Figure 6:**
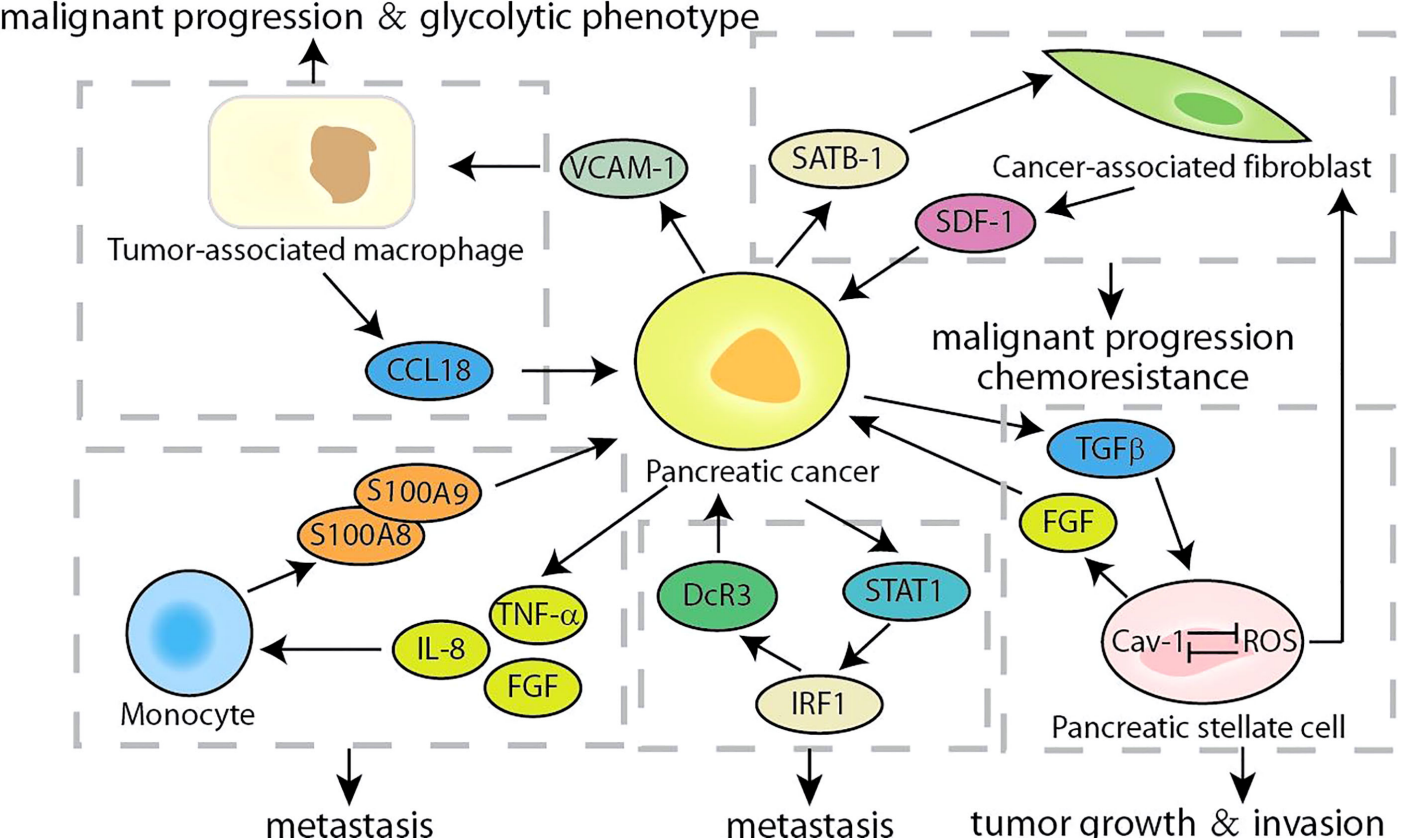
Summary of the crosstalk of cells related feedback loops in pancreatic cancer.

The hallmark of pancreatic cancer is the complex and variable tumor microenvironment, which includes cancer-associated fibroblasts (CAF), tumor-associated macrophages (TAM), monocytes, and the extracellular matrix surrounding cancer cells (80, 81). The secretion of cytokines makes it possible to establish crosstalk between tumor cells and the surrounding microenvironment, which were crucial for promoting the progress of pancreatic cancer. According to reports, SDF-1 secreted by CAF stimulated the malignant progression of pancreatic cancer and gemcitabine-resistance, which is partly caused by the paracrine regulation of SATB-1 in pancreatic cancer (**Figure 6**). The expression of SATB-1 in pancreatic cancer was regulated by SDF-1 secreted by CAF, which helps to maintain the characteristics of CAF and form a mutually beneficial feedback loop (54). CXCR4 was the key bridge of the SDF-1/STAB-1 loop, and both CXCR4 inhibitors AMD3100 and BL-8040 exhibited encouraging results in PC therapy (55, 82). Similarly, Chen et al. found that CCL18 secreted by TAMs facilitated malignant progression and the glycolytic phenotype in pancreatic cancer, which is partly caused by the paracrine induction of VCAM-1 in pancreatic cancer (83). In turn, VCAM-1 induced pancreatic cancer cells to produce lactic acid and aerobic glycolysis, which promoted the TAM-like phenotype of macrophages to form a positive feedback loop. The VCAM-1-specific monoclonal antibody used by Lee et al. may block the signaling loop between TAMs and pancreatic cancer cells, which was beneficial to the clinical treatment of pancreatic cancer (84). Although accounting for partial proportion of the pancreatic cancer tumor microenvironment, monocytes also show considerable roles. The S100A8/S100A9 secreted by monocytes regulated the behavior of pancreatic cancer cells by causing a pre-metastasis cascade related to cancer spread (56). Pancreatic cancer cells stimulated by S100A8 and S100A9 increased the secretion of pro-inflammatory cytokines IL-8, TNF-α and FGF. In contrast, conditioned media and cytokines derived from cancer cells upregulated the expression of S100A8 and S100A9 in monocytes. Accumulated evidence indicated that the expression of decoy receptor 3 (DcR3), a secreted molecule, was positively correlated with the malignant process of pancreatic cancer and poor survival (57, 85). The S100 neutralizing antibody, peptibodies and peptide-Fc fusion proteins appeared to represent a promising approach to impede the loop of monocytes and pancreatic cancer cells, which had been shown to reduce tumor burden in varieties of cancer models (86). Recent studies have shown that DcR3 promotes the phosphorylation of STAT1 and leads to a sharp increase in IRF1. DcR3/STAT1/IRF1 formed a positive feedback loop to amplify the transcriptional expression of DcR3, providing a potential therapeutic target for pancreatic cancer (58). It was found that triptolide can induce pancreatic cancer cell apoptosis by down-regulating DcR3 expression, and had the potential as an effective drug for pancreatic cancer (59). Caveolin-1 (Cav-1) is the main structural component of the cave, and its disorder occurs in pancreatic cancer and is related to the patient’s prognosis (60). In pancreatic stellate cells, Cav-1 knockdown promoted the production of ROS, and the production of ROS further reduces Cav-1 expression. The positive feedback transduction of Cav-1-ROS signal promoted the growth of pancreatic cancer and induced matrix-tumor metabolic coupling (61). And FGF secreted by pancreatic stellate cells stimulated cancer cells to produce TGFβ, and one of the main functions of TGFβ was to promote the activation of PSCs and converted them into CAF, forming a positive feedback loop to drive the invasion of pancreatic cancer cells (87, 88). There were two main types of targeted drugs for FGFR. One was multi-tyrosine kinase inhibitors, which mainly included Ponatinib, Dovitinib, and nintedanib; the other was FGFR selective inhibitors, which mainly included PD173074, AZD4547, and BGJ398 (89). At present, these drugs had entered clinical or preclinical studies, and it was conceivable that targeting and interfering with the FGFR loop would benefit pancreatic cancer patients. Growing evidence highlighted the importance of understanding the multifaceted roles of complex tumor microenvironment signaling feedback loops in pancreatic cancer. Therefore, future approaches should prioritize intervention or blockade of these targets, and combinatorial strategies that simultaneously target multiple feedback loops of the tumor microenvironment may also be successful.

## Negative Feedback Loop

**Figure 7:**
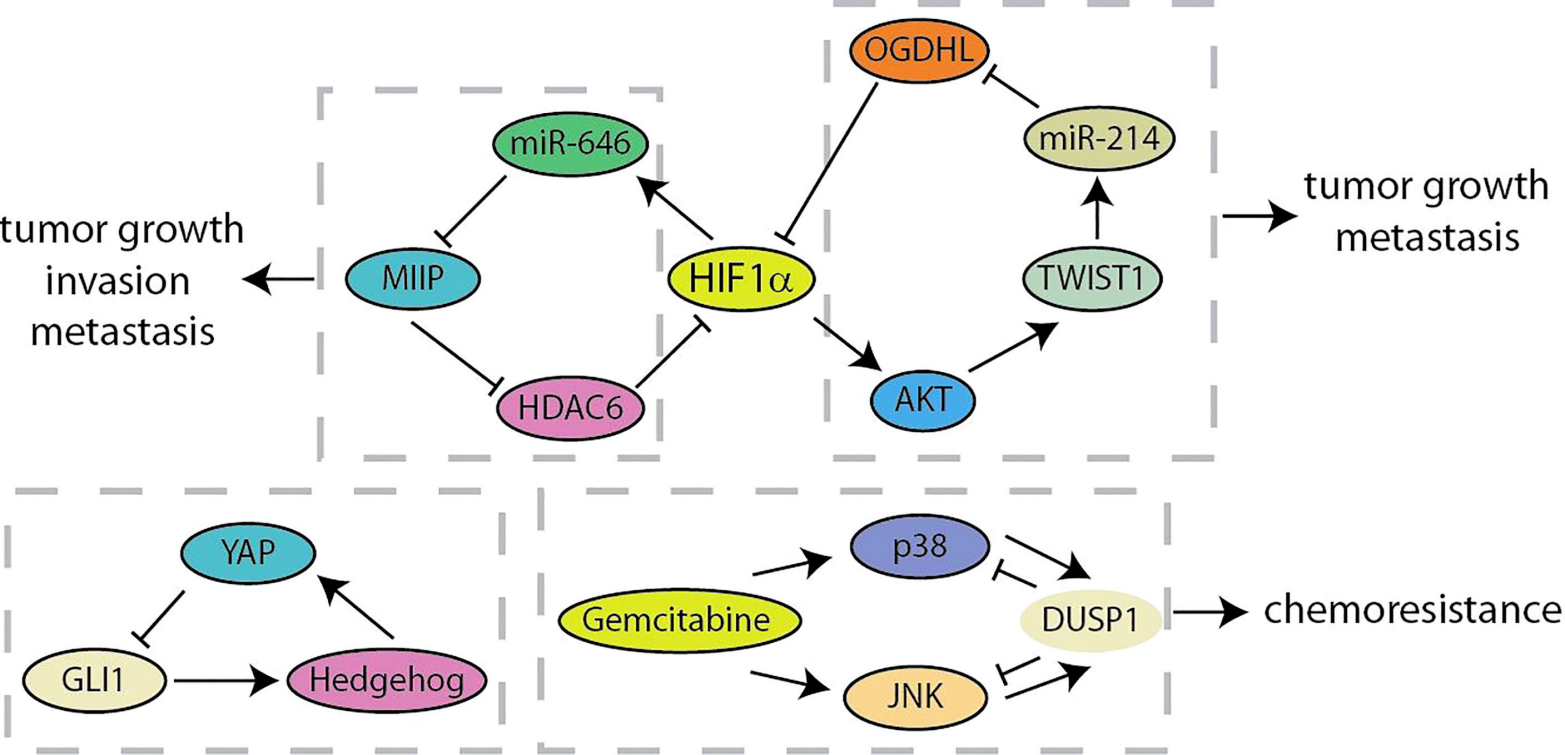
Summary of negative feedback loops in pancreatic cancer.

The negative feedback loop effectuates adaptation to external or internal signals, robustness to noise, and dynamic plasticity of cancer cells (18, 90). Migration and Invasion Inhibitory Protein (MIIP) has recently been identified as an inhibitor of tumor development and constitutes a negative feedback loop with HIF1α in pancreatic cancer (**Figure 7**) (91). MIIP expression was regulated by HIF1α-induced miR-646 activation at the transcriptional level, and MIIP conversely inhibited the deacetylase activity of HDAC6, resulting in the promotion of acetylation and degradation of HIF1α. Liu et al. have found the prognostic value of the combination of oxoglutarate dehydrogenase like (OGDHL), TWIST1 and miR-214 in predicting pancreatic cancer patients (92). TWIST1 upregulates miR-214 and induces the suppression of OGDHL. What is more, OGDHL decreases TWIST1 expression by promoting ubiquitin-mediated degradation of HIF1α and regulating the AKT signaling. Recent results identified a feedback crosstalk between YAP and Hedgehog at the transcription level (93). GLI transcription factors are negatively controlled by YAP binding to impede Hedgehog signaling. In contrast, Hedgehog improves YAP activity by post-transcriptional mechanisms in pancreatic cancer. As a key negative inhibitor of MAPK, DUSP1 is transcriptionally upregulated when JNK/p38MAPK is activated by gemcitabine. Gemcitabine-mediated “dual up-regulation” is conducive to the formation of a negative feedback loop, thereby weakening its beneficial effects on stress pathways and apoptosis in the treatment of pancreatic cancer (94). Compared with positive feedback loops, there are currently fewer studies on negative feedback loops in pancreatic cancer, which means that more researchers are needed to invest in this area (**Table 1**).

## Intervened Feedback Loops and Treatment in Pancreatic Cancer

Currently, the involvement of feedback loops in regulating the malignant evolution of pancreatic cancer has achieved remarkable progress in preclinical studies. These findings provide important lessons and showed that the validation of drugs or small molecules targeting the feedback loops would facilitate the obtaining of strategies to regulate key targets and improve the clinical efficacy of single-drug treatment (**Table 2**). The PI3K/Akt pathway is continuously activated in pancreatic cancer, and mTOR, the target of rapamycin, is a crucial mediator of its signal transduction (95). In a phase II study of inhibition of mTOR in advanced pancreatic cancer, prolonged treatment with mTOR inhibitors could enhance the insulin receptor substrate-PI3K interaction, and paradoxically promote the phosphorylation of AKT to form a negative feedback loop (10). The synergistic treatment of erlotinib and rapamycin significantly inhibited activated-AKT by the inhibition of rapamycin and showed better anti-tumor activity in preclinical models. It is foreseeable that the collaborative strategies for feedback loops would be more widely applied in the clinical treatment of pancreatic cancer. The KRAS-E2F1-ILK-hnRNPA1 regulatory loop was found recently, which provided a drug-able target ILK to inhibit the oncogenic KRAS signal from the therapeutic point of view. T315, an ILK inhibitor, was found to decrease the expression of KRAS, E2F1, ILK and hnRNPA1 in a dose-dependent manner and significantly inhibited the viability of pancreatic cancer cells (21, 22). ODC activity, polyamine biosynthesis and spermidine production were inhibited by DFMO and GC7, which are required for eIF5A hypusination and prevent pancreatic cancer progression (96). Mechanismly, the inhibition of eIF5A hypusination by DFMO and GC7 effectively attenuated the feedback expression of KRas, and significantly increased the sensitivity of KRas-driven PC cells to Ras-Erk signaling inhibitors (23). Recent studies indicated that treatment with AG490, a JAK2/STAT3 inhibitor, reduced the survival rate of pancreatic cancer cells that strongly relay on mutp53 expression (26). Phosphorylation of STAT3 inhibited by AG490 would down-regulate mutp53, which in turn decreased HSP90 expression. Geldanamycin, an HSP90 inhibitor, significantly reduced the expression of mutp53 in pancreatic cancer, and geldanamycin surprisingly reduced STAT3 phosphorylation. It was suggested that HSP90 may in turn maintain STAT3 phosphorylation, which is a positive feedback loop that critical to the stability of mutp53. Despite the promising effects of NF-κB signaling pathway inhibitors in inhibiting the progression of a variety of cancers, lack of specific indicators for cancer treatment is one of the most critical dilemmas limiting the treatment of NF-κB signaling inhibitors (97). Chen et al. found that treatment with JSH-23, a NF-κB inhibitor, blocked the PLAT1-NF-κB-E2F1 signaling loop and effectively inhibited the tumorigenesis of pancreatic cancer overexpressing PLACT1 *in vivo (* 31). Likely, HGF/Met signaling inhibitors have shown anti-tumor effects in preclinical animal models, but the single treatment of Met inhibitor had only moderate clinical benefit in treating cancers with abnormal HGF/Met signaling (98). In the study of mechanism of the resistance of pancreatic cancer cells to Met-inhibition, the synergistic treatment with FOXM1 inhibitor TST and Met inhibitor PHA-665752 significantly hindered the growth of resistant cells *in vitro* or vivo, and the obstacle of HGF/Met-FOXM1 loop by the combination of TST and PHA-665752 was more effective than either inhibitor alone (9). Thus, research on the molecular biology mechanisms to elucidate how feedback loops modulate the progression of pancreatic cancer is of great importance for feedback control and crosstalk between signal pathways by successful drug selection and synergistic treatment.

**Table 2:** Intervened feedback loops and treatments in pancreatic cancer.

Drugs	Key molecules	Feedback loop modulation	References
Erlotinib, Rapamycin	mTOR/PI3K/Akt	Negative	(10)
T315	KRAS-E2F1-ILK-hnRNPA1	Positive	(21, 22)
DFMO, GC7	KRAS/eIF5A/PEAK1	Positive	(23, 88)
AG490, Geldanamycin	mutant-P53/STAT3/HSP90	Positive	(25, 26)
JSH-23	PLACT1/hnRNPA1/IκBα/E2F1	Positive	(31)
TST, PHA-665752	HGF/Met/FOXM1	Positive	(9, 90)

## Discussion

Pancreatic cancer remains a highly lethal malignant cancer with limited options for effective therapy in the past five years and even in the future. Pancreatic cancer has an extremely complex signal regulation network, which mediates the initiation, malignant progression, metastasis and recurrence. Discovery, elucidation and targeted regulation of the signal regulation network within pancreatic cancer can provide potential new methods for cancer treatment. However, in view of the complex and fickle characteristics of pancreatic cancer, the factors and concise modulation mechanisms for activation or inactivation of internal signaling pathways remain poorly understood. The feedback loop regulation of signals has dual effects on the progression of pancreatic cancer. On the one hand, the upstream and downstream crucial molecules in the circuit regulate each other to form a signal amplification cascade, which contributes to the development of cancer by inducing cancer cells proliferation, metastasis and drug-resistance. On the other hand, preclinical and clinical studies have indicated that the feedback loop may inhibit the development of cancer cells to a certain extent in some cases. In conclusion, the relationship of feedback loops with pancreatic cancer is only partially understood, and future research needs to further explore and clarify detailed regulating mechanisms of feedback loops in pancreatic cancer that can be applied to target significant oncogenic signals to achieve the greatest benefit of treatment.

The analysis of feedback loops and crosstalk between signaling pathways was critical for the success of drug clinical therapy in pancreatic cancer, as feedback loops greatly altered drug effectiveness and sensitivity. Multiple lines of evidence now spanning different cellular contexts and cancer types suggested that feedback loops may lead to unexpected upregulation or loss of key signals. This either directly rendered the drug ineffective or the activation of alternative pro-survival pathways that detract from the expected efficacy of anticancer drugs, which seemed to explain the disappointing clinical results. Researchers should draw lessons from these results to design new drug combinations or other more comprehensive approaches that counteract changes in feedback loop signals to achieve therapeutic goals. It was true that pancreatic cancer had numerous feedback loops that cannot be adjusted all. Merely intervening in a feedback loop, while potentially effective, was likely to be insufficient. Therefore, it was necessary to intervene in the basic signaling circuits that control tumor growth and spread, so as to achieve therapeutic purposes. Of course, the role of other feedback loops could not be ignored.

We summarized the discovered feedback loops of pancreatic cancer resistance to therapy, but it was no doubt that there are still more feedback signals hidden in numerous protein-protein interactions. How to tap more potential feedback loops was an appealing topic. These possibilities were not without clues, and we could look for potential oncogenic feedback signals based on the underlying functional regulatory network. For example, transcription factors were significantly activated in pancreatic cancer, so the enzymes that potentially regulated their deubiquitination are significantly up-regulated, which in turn may be a feedback loop leading to amplification of oncogenic signals. Transcription factors needed to transcriptionally regulate downstream genes in specific substructures, and it was questionable whether their transporters formed the criminal alliance with the transcription factor. The regulatory network and mechanism of pancreatic cancer were complex, but finding more feedback signals was worthy of more investment by researchers.

Currently, preclinical findings and clinical studies applying intervention strategy of feedback loop to pancreatic cancer therapies have exhibited encouraging results, which has instigated a demand for novel feedback loop modulators with higher efficacy and safety. The further exploration and exploitation of feedback loop modulators is expected to facilitate the practicality of using these therapeutic approaches and offer more benefits to pancreatic cancer patients.

## Author Contributions

JY and XZ designed the study. WG contributed to discussions of its content. HS drew all figures and tables. JY and LX wrote the manuscript and reviewed. JY and XZ edited the manuscript before submission. All authors contributed to the article and approved the submitted version.

## Funding

This study was supported by grants from the Zhejiang health committee (WKJ-ZJ-2136, 2021ZH003), Hangzhou science and technology commission (202004A14) and The Construction Fund of Key Medical Disciplines of Hangzhou (OO20190001).

## Conflict of Interest

The authors declare that the research was conducted in the absence of any commercial or financial relationships that could be construed as a potential conflict of interest.

## Publisher’s Note

All claims expressed in this article are solely those of the authors and do not necessarily represent those of their affiliated organizations, or those of the publisher, the editors and the reviewers. Any product that may be evaluated in this article, or claim that may be made by its manufacturer, is not guaranteed or endorsed by the publisher.
